# Diagnosis and Treatment of Bacterial Pneumonia in Critically Ill Patients with COVID-19 Using a Multiplex PCR Assay: A Large Italian Hospital’s Five-Month Experience

**DOI:** 10.1128/Spectrum.00695-21

**Published:** 2021-11-10

**Authors:** Brunella Posteraro, Venere Cortazzo, Flora Marzia Liotti, Giulia Menchinelli, Chiara Ippoliti, Giulia De Angelis, Marilena La Sorda, Gennaro Capalbo, Joel Vargas, Massimo Antonelli, Maurizio Sanguinetti, Gennaro De Pascale, Teresa Spanu

**Affiliations:** a Dipartimento di Scienze Biotecnologiche di Base, Cliniche Intensivologiche e Perioperatorie, Università Cattolica del Sacro Cuore, Rome, Italy; b Dipartimento di Scienze Mediche e Chirurgiche, Fondazione Policlinico Universitario A. Gemelli IRCCS, Rome, Italy; c Dipartimento di Scienze di Laboratorio e Infettivologiche, Fondazione Policlinico Universitario A. Gemelli IRCCS, Rome, Italy; d Hospital Health Management, Fondazione Policlinico Universitario A. Gemelli IRCCS, Rome, Italy; e Dipartimento di Scienze dell’Emergenza, Anestesiologiche e della Rianimazione, Fondazione Policlinico Universitario A. Gemelli IRCCS, Rome, Italy; Montefiore Medical Center and Albert Einstein College of Medicine

**Keywords:** bacterial pneumonia, COVID-19, diagnosis, FilmArray panel, treatment

## Abstract

Bacterial pneumonia is a challenging coronavirus disease 2019 (COVID-19) complication for intensive care unit (ICU) clinicians. Upon its implementation, the FilmArray pneumonia plus (FA-PP) panel’s practicability for both the diagnosis and antimicrobial therapy management of bacterial pneumonia was assessed in ICU patients with COVID-19. Respiratory samples were collected from patients who were mechanically ventilated at the time bacterial etiology and antimicrobial resistance were determined using both standard-of-care (culture and antimicrobial susceptibility testing [AST]) and FA-PP panel testing methods. Changes to targeted and/or appropriate antimicrobial therapy were reviewed. We tested 212 samples from 150 patients suspected of bacterial pneumonia. Etiologically, 120 samples were positive by both methods, two samples were culture positive but FA-PP negative (i.e., negative for on-panel organisms), and 90 were negative by both methods. FA-PP detected no culture-growing organisms (mostly Staphylococcus aureus or Pseudomonas aeruginosa) in 19 of 120 samples or antimicrobial resistance genes in two culture-negative samples for S. aureus organisms. Fifty-nine (27.8%) of 212 samples were from empirically treated patients. Antibiotics were discontinued in 5 (33.3%) of 15 patients with FA-PP-negative samples and were escalated/deescalated in 39 (88.6%) of 44 patients with FA-PP-positive samples. Overall, antibiotics were initiated in 87 (72.5%) of 120 pneumonia episodes and were not administered in 80 (87.0%) of 92 nonpneumonia episodes. Antimicrobial-resistant organisms caused 78 (60.0%) of 120 episodes. Excluding 19 colistin-resistant Acinetobacter baumannii episodes, AST confirmed appropriate antibiotic receipt in 101 (84.2%) of 120 episodes for one or more FA-PP-detected organisms. Compared to standard-of-care testing, the FA-PP panel may be of great value in the management of COVID-19 patients at risk of developing bacterial pneumonia in the ICU.

**IMPORTANCE** Since bacterial pneumonia is relatively frequent, suspicion of it in COVID-19 patients may prompt ICU clinicians to overuse (broad-spectrum) antibiotics, particularly when empirical antibiotics do not cover the suspected pathogen. We showed that a PCR-based, culture-independent laboratory assay allows not only accurate diagnosis but also streamlining of antimicrobial therapy for bacterial pneumonia episodes. We report on the actual implementation of rapid diagnostics and its real-life impact on patient treatment, which is a gain over previously published studies on the topic. A better understanding of the role of that or similar PCR assays in routine ICU practice may lead us to appreciate the effectiveness of their implementation during the COVID-19 pandemic.

## INTRODUCTION

Since the global spread of severe acute respiratory syndrome coronavirus 2 (SARS-CoV-2), the etiological agent of human coronavirus disease 2019 (COVID-19), bacterial pneumonia, mostly ventilator-associated pneumonia (VAP), has been reported as a relatively frequent COVID-19 complication in critically ill patients, such as those admitted to the intensive care unit (ICU) (1–7).

Whenever feasible (8), sampling of lower respiratory tract (LRT) secretions (i.e., obtaining bronchoalveolar lavage [BAL] fluid or endotracheal aspirate [ETA] samples) is crucial to provide detection of organisms coupled with assessment of their bacterial loads in COVID-19 patients who develop VAP or non-VAP hospital-acquired pneumonia (HAP) during the ICU stay (9). In this context, newer multiplex PCR-based or array-based multipathogen and, conditionally, antimicrobial resistance (AMR) gene detection assays helped to diagnose bacterial infection (either secondary or concomitant to SARS-CoV-2 infection) in ICU patients with COVID-19 (1, 5, 10, 11). However, the impact of these assays on antibiotic use has scarcely been explored.

In September 2020, when the second wave of the SARS-CoV-2 pandemic was raging in Italy, we implemented the multiplexed, semiquantitative FilmArray pneumonia plus (FA-PP) panel (BioFire, Salt Lake City, UT, USA) assay (12–14) for routine clinical use at our institution (a large Italian university hospital). The goal of this implementation was to avoid unnecessarily or inappropriately administered antimicrobials to prevent infectious complications in patients with COVID-19. Here, we report on the FA-PP panel testing of LRT (BAL fluid or ETA) samples from ICU-admitted COVID-19 patients over a 5-month period (September 2020 to March 2021). In particular, we assessed the agreement of FA-PP panel results with those of culture-based standard-of-care (SoC) methods and the impact of FA-PP panel results on antimicrobial therapy management in patients with bacterial pneumonia.

## RESULTS

### Diagnosis of bacterial pneumonia using the FA-PP panel.

We tested 212 (82 BAL fluid and 130 ETA) LRT samples from 150 COVID-19 patients hospitalized in our ICU using both standard of care (SoC; including bacterial culture, identification, and AMR assessment) and FA-PP panel testing methods (Fig. S1 in the supplemental material). In total, 120 samples were positive by both methods, two samples were culture positive but FA-PP panel negative (i.e., negative for on-panel organisms), and 90 were negative by both methods. The two culture-positive samples grew organisms not targeted by the FA-PP panel (i.e., off-panel organisms), including Stenotrophomonas maltophilia and Citrobacter koseri (1 sample) or S. maltophilia alone (1 sample).

Table 1 shows the qualitative agreement of results for 16 microbial targets (13 bacterial species and 3 AMR genes, corresponding to 202 bacterial organisms in total) detected by the FA-PP panel along with results by culture (and/or the AMR assessment for 47 organisms), which was used as the reference method. The positive percent agreement (PPA) values were 100% for all evaluable targets. Among bacterial species, negative percent agreement (NPA) values ranged from 96.4% for Staphylococcus aureus (155/161) to 100% for the Acinetobacter calcoaceticus-*baumannii* complex (159/159), Klebsiella aerogenes (207/207), Klebsiella pneumoniae group (189/189), and Proteus spp. (210/210). Among AMR genes, NPAs were 100% for CTX-M (200/200) and KPC (202/202) and 98.9% for *mecA*/-*C* and MREJ (*mec* right-extremity junction) (185/187). As shown in Table 1, 22 of 202 FA-PP panel-detected organisms that did not grow in culture were from 19 samples, of which 18 samples each had one additional detected organism (i.e., only detected by the FA-PP panel) and 1 sample had four additional detected organisms. Furthermore, the FA-PP panel detected additional (*mecA*/-*C* and MREJ) AMR genes in two samples with S. aureus organisms that did not grow in culture (Table 1).

**TABLE 1:** Comparison between the FilmArray pneumonia plus panel and standard-of-care reference testing results for LRT samples from COVID-19 patients with bacterial pneumonia^*a*^

Microbial target	No. positive by FA-PP and SoC/no. positive by SoC	PPA (%) (95% CI)	No. negative by FA-PP and SoC/no. negative by SoC	NPA (%) (95% CI)	No. positive only by FA-PP for samples from patients who were^*b*^:	
					Under antimicrobial therapy	Not under antimicrobial therapy
Bacterial species						
Acinetobacter calcoaceticus-*baumannii* complex	53/53	100 (93.2–100)	159/159	100 (97.7–100)		
Enterobacter cloacae complex	4/4	100 (39.8–100)	206/207	99.5 (97.4–100)	1	
Escherichia coli	15/15	100 (78.2–100)	195/196	99.5 (97.2–100)	1	
Haemophilus influenzae	2/2	100 (15.9–100)	208/209	99.5 (97.4–100)		1
Klebsiella aerogenes	5/5	100 (47.8–100)	207/207	100 (98.2–100)		
Klebsiella oxytoca	2/2	100 (15.8–100)	204/207	98.6 (95.9–99.7)	1	2
Klebsiella pneumoniae group	23/23	100 (85.2–100)	189/189	100 (98.1–100)		
Proteus spp.	2/2	100 (15.9–100)	210/210	100 (98.3–100)		
Pseudomonas aeruginosa	19/19	100 (82.4–100)	185/189	97.9 (94.8–99.4)	4	
Serratia marcescens	6/6	100 (54.1–100)	200/203	98.5 (95.8–99.7)	2	1
Staphylococcus aureus	45/45	100 (92.1–100)	155/161	96.4 (92.3–98.7)	5	1
Streptococcus agalactiae	0/0	NC	208/210	99.1 (96.6–99.9)		2
Streptococcus pneumoniae	4/4	100 (39.8–100)	206/207	99.5 (97.4–100)		1

Total species	180/180	100 (98.0–100)	2,532/2,554	99.2 (98.7–99.5)	14	8

Antimicrobial resistance genes						
CTX-M	12/12	100 (73.5–100)	200/200	100 (98.2–100)		
KPC	10/10	100 (69.2–100)	202/202	100 (98.2–100)		
*mecA*/-*C* and MREJ^*c*^	23/23	100 (85.2–100)	185/187	98.9 (96.2–99.9)	2	

Total genes	45/45	100 (92.1–100)	587/589	99.7 (98.8–100)	2	

a Excluding off-panel organisms, FA-PP panel testing results were compared with those obtained by the SoC testing method that was used as the reference method. This method included bacterial identification, antimicrobial susceptibility testing, and (only for antimicrobial resistance genes) PCR-sequencing analysis, which were performed on the microbial species isolated in culture. FA-PP, FilmArray pneumonia plus panel; SoC, standard of care; LRT, lower respiratory tract; COVID-19, coronavirus disease 2019; PPA, positive percent agreement; NPA, negative percent agreement; CI, confidence interval; NC, not calculated.

b The 22 species only detected by the FA-PP panel were from 19 LRT samples, of which 18 samples (13 monomicrobial and 5 polymicrobial by culture) each had one additional organism and 1 sample (polymicrobial by culture) had four additional organisms.

c The FA-PP panel identifies methicillin-resistant S. aureus (MRSA) based on the detection of *mecA*/-*C* and MREJ (*mec* right-extremity junction).

**FIG 1:**
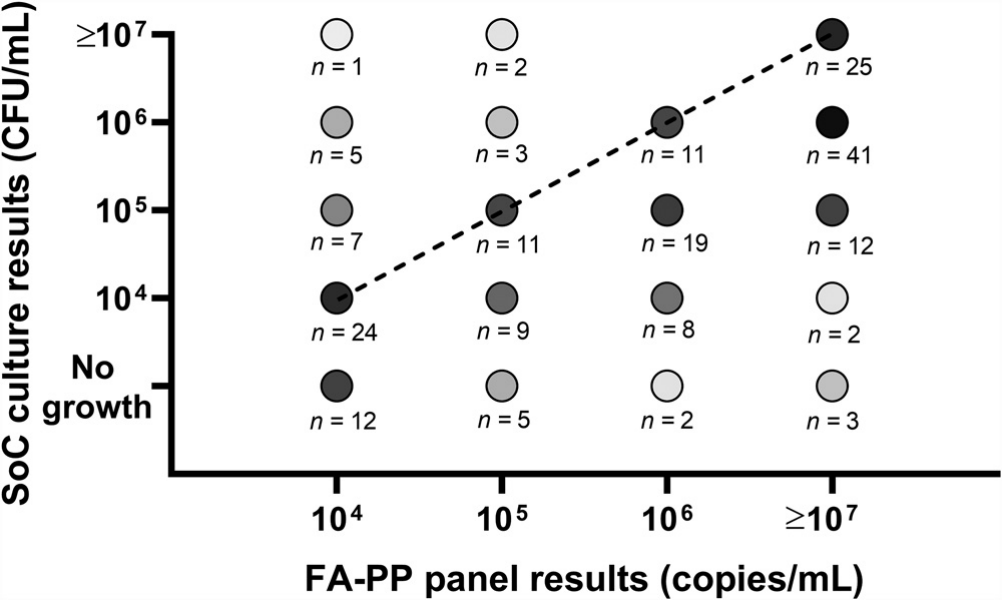
Quantitative result agreement between the FilmArray pneumonia plus (FA-PP) panel and standard-of-care (SoC) culture testing methods for 202 bacterial organisms detected in bronchoalveolar lavage (BAL) fluid or endotracheal aspirate (ETA) samples from ICU patients. All but 22 (which did not grow in culture) organisms were detected at or above the 1 × 10^4^ CFU/ml (BAL fluid sample) or 1 × 10^5^ CFU/ml (ETA sample) thresholds for clinically relevant quantification by both methods. The diagonal dashed line connects dots that represent bacterial organisms with fully concordant results (71/202; 35.1%), whereas dots above or below the line represent bacterial organisms with SoC culture loads that exceeded (18/202, 8.9%) or did not exceed (113/202, 56.0%), respectively, those of the FA-PP panel by ≥1 log_10_. Shades of gray are used to depict the different numbers of samples corresponding to each dot.

Fig. 1 shows the quantitative agreement between the results for the 202 bacterial organisms detected by the FA-PP panel and the culture results. Of these organisms, 83 were detected as ≥10^7^ genome copies/ml (25 [30.1%] of which were quantified in culture at ≥10^7^ CFU/ml), and 40 were detected as 10^6^ genome copies/ml (11 [27.5%] of which were quantified in culture at 10^6^ CFU/ml). Another 30 organisms were detected as 10^5^ genome copies/ml (11 [36.7%] of which were quantified in culture at 10^5^ CFU/ml), and 49 were detected as 10^4^ genome copies/ml (24 [49.0%] of which were quantified in culture at 10^4^ CFU/ml). In the aforementioned (BAL fluid) sample with an additional four organisms, the FA-PP panel detection values were ≥10^7^ (four organisms, two of which grew in culture), 10^5^ (one organism), and 10^4^ (one organism) genome copies/ml. Fourteen of 22 organisms (mostly S. aureus [including 2 *mecA*/-*C* and MREJ positive] or Pseudomonas aeruginosa) only detected by the FA-PP panel were in samples from patients empirically treated with antibiotics (Table 1), and 8 of 14 organisms had FA-PP panel detection values of 10^4^ genome copies/ml. Conversely, 8 of 22 organisms were detected in samples from patients not empirically treated with antibiotics (Table 1), and 4 of 8 organisms belonged to difficult-to-culture bacterial species like Streptococcus agalactiae, Haemophilus influenzae, or Streptococcus pneumoniae.

According to the FA-PP results, as detailed below, antibiotics were continued (e.g., linezolid for two aforementioned *mecA*/-*C*- and MREJ-positive S. aureus organisms) and/or modified in the case of samples collected from patients under empirical antimicrobial therapy or were initiated in the case of samples collected from patients not under empirical antimicrobial therapy.

### Treatment of bacterial pneumonia using the FA-PP panel.

The results of FA-PP panel testing were immediately accessible to ICU clinicians for antimicrobial therapy management. Of 212 LRT samples, 59 (27.8%), 15 and 44 of which were negative or positive, respectively, on the FA-PP panel, were from patients empirically treated with antibiotics. In 5 (33.3%) of the 15 patients, antibiotics were discontinued, and in 39 (88.6%) of the 44 patients, antibiotics were escalated/deescalated. Overall, antibiotics were not administered in 80 (87.0%) of 92 samples with a negative FA-PP panel result, whereas antibiotics were initiated in 87 (72.5%) of 120 samples with a positive FA-PP panel result.

**FIG 2:**
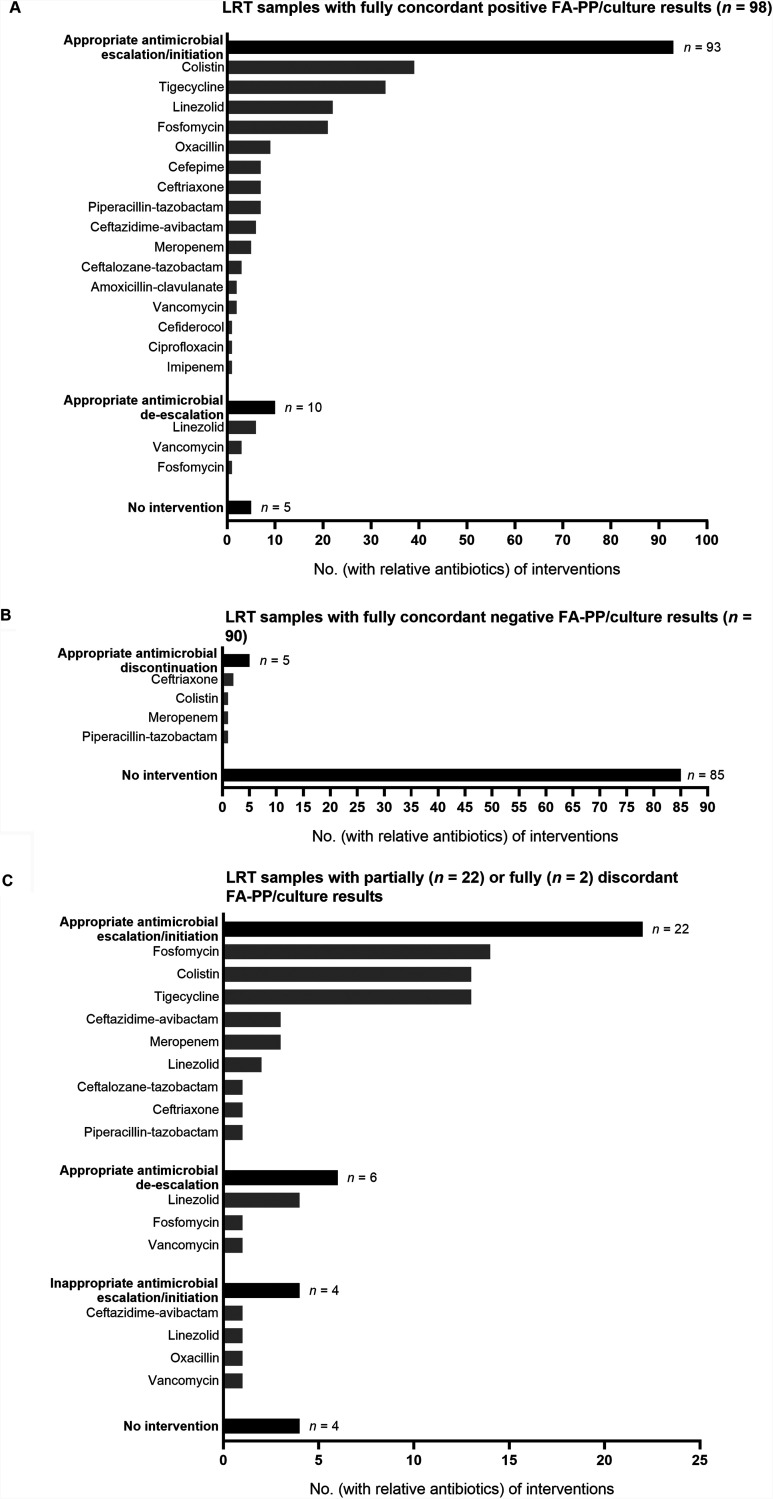
Overview of antimicrobial therapy interventions performed according to FilmArray pneumonia plus (FA-PP) panel results. Interventions were stratified into three relevant groups based on the full concordance of positive (A) or negative (B) FA-PP testing results or the partial or full discordance (C) of FA-PP testing results, respectively, with the results by standard-of-care (SoC) culture for the lower respiratory tract (LRT) samples tested. For each LRT sample, the type(s) of interventions and the antibiotics involved (one or more per intervention) are reported, together with whether the interventions resulted in appropriate antimicrobial escalations/initiations (93 in panel A and 22 in panel C) or deescalations (10 in panel A and 6 in panel C), appropriate antimicrobial discontinuations (5 in panel B), and inappropriate antimicrobial escalations/initiations (4 in panel C). Cases with no antimicrobial therapy interventions (5 in panel A, 85 in panel B, and 4 in panel C) are reported as well.

Fig. 2 shows the FA-PP result-based therapeutic interventions for the study patients stratified by groups of samples according to whether FA-PP and culture results were fully concordant positive (98/212, 46.2%), fully concordant negative (90/212, 42.4%), partially discordant (22/212, 10.4%), or fully discordant (2/212, 0.9%). For 98 samples (Fig. 2A), the results for one or more FA-PP/culture-detected organisms were fully concordant between the methods. Accordingly, antimicrobial therapy was appropriately initiated/escalated in 93 cases and deescalated in 10 cases, with initiation/escalation and deescalation occurring simultaneously in 10 cases. For 90 samples (Fig. 2B), there were no FA-PP-/culture-detected organisms, and the results were fully concordant between the methods. Accordingly, antimicrobial therapy was appropriately discontinued in five cases. For 22 samples (Fig. 2C), partially discordant FA-PP/culture results included those for 19 samples with organisms detected by both methods together with organisms only detected by the FA-PP panel and 3 samples with organisms detected by both methods together with organisms only detected by culture. Two other samples (Fig. 2C) had fully discordant FA-PP/culture results because they were each culture positive for one or more organisms not included in the FA-PP panel. Accordingly, antimicrobial therapy was appropriately initiated/escalated in 22 cases and deescalated in 6 cases, with initiation/escalation and deescalation occurring simultaneously in six cases. Furthermore (Fig. 2C), in 4 of 19 samples with additional FA-PP panel-detected organisms, interventions resulted in (inappropriately) initiating linezolid (1 sample), oxacillin (1 sample), or vancomycin (1 sample) to cover S. aureus organisms (three in total) or escalating ceftazidime-avibactam to cover a P. aeruginosa organism (1 sample). In four of the remaining five samples with additional culture-detected organisms (including the two samples only positive for off-panel organisms), interventions did not occur at the time of FA-PP results. This situation occurred regardless of an empirical antimicrobial therapy that did not cover S. maltophilia organisms (2 samples) or was not administered (one sample positive for both S. maltophilia and C. koseri and one sample positive for Hafnia alvei).

Regarding the 120 interventions in total (Fig. 2), the mean (±standard deviation [SD]) time to appropriate antimicrobial therapy, based on FA-PP results, was 6.3 ± 6.5 h. Only considering the interventions for samples with fully concordant positive or negative FA-PP/culture results, the time (6.4 ± 6.6 h) would have been delayed by an average of 72 h if culture results had been used to guide the patients’ antimicrobial treatment.

Substantially, therapeutic interventions included 52 of 53 A. baumannii episodes, for which patients received colistin/tigecycline combinations (16 episodes), colistin/tigecycline/fosfomycin combinations (30 episodes), colistin alone (5 episodes), or cefiderocol (1 episode). Later (at least 2 days later), available antimicrobial susceptibility testing (AST) results showed that all A. baumannii organisms had a carbapenem-resistant phenotype (confirmed by *bla*_oxa-23_ gene detection using PCR-sequencing analysis) and that 19 (35.9%) of 53 A. baumannii organisms had a colistin-resistant phenotype. With respect to S. aureus episodes, initiations or escalations occurred in 39 (76.5%) of 51 episodes, for which patients received linezolid (24 methicillin-resistant S. aureus [MRSA] episodes and 1 methicillin-susceptible [MSSA] episode), oxacillin (9 MSSA episodes), amoxicillin-clavulanate (2 MSSA episodes), or vancomycin (2 MSSA episodes and 1 MRSA episode). In another MSSA episode, linezolid was deescalated to oxacillin.

### Characteristics of bacterial pneumonia episodes included in the study.

In total (Table 2), we studied 122 bacterial pneumonia episodes, with 120 of them being positive for one (65 episodes), two (38 episodes), three (10 episodes), four (5 episodes), five (1 episode), or six (1 episode) on-panel organisms. In particular, 97, 16, 3, or 1 patient had a first, second, third, and fourth episode, respectively. For patients with multiple episodes, the time elapsing between successive episodes ranged from 7 days (one patient was initially infected by cephalosporin-susceptible, CTX-M-negative Escherichia coli and was then infected by cephalosporin-resistant, CTX-M-positive E. coli) to 60 days (one patient was initially infected by colistin-resistant Acinetobacter baumannii and was then infected by colistin-resistant A. baumannii plus Serratia marcescens). Of 122 episodes, 53 (43.4%) were caused by A. baumannii organisms, and 51 (41.8%) were caused by S. aureus. Organisms (listed in order of frequency) supposed to stem from the oropharyngeal microbiota (S. pneumoniae, H. influenzae, and S. agalactiae) were found almost exclusively in non-VAP episodes (six in total). Notably, H. influenzae was one of the causative pathogens from one (polymicrobial) VAP episode, whereas S. pneumoniae was found alone (one episode) or together with other (community or nosocomial) pathogens in four of six non-VAP episodes.

**TABLE 2:** Characteristics of bacterial pneumonia episodes detected or not detected by the FilmArray pneumonia plus panel in 97 mechanically ventilated patients with COVID-19^*a*^

Episode	No. (%)
All episodes	122 (100)
On-panel organism caused^*b*^	120 (98.4)
Off-panel organism caused^*c*^	2 (1.6)
First episodes	97 (79.5)
VAP episodes (i.e., occurring at >48 h of ventilation)	89 (72.9)
Non-VAP episodes (i.e., occurring at ≤48 h of ventilation)	33 (27.1)
Monomicrobial infections	64 (52.5)
Polymicrobial infections	58 (47.5)
Acinetobacter baumannii infections	53 (43.4)
Staphylococcus aureus infections	51 (41.8)
Antimicrobial-resistant infections^*d*^	78 (63.9)
Episodes with concurrent bacteremia^*e*^	31 (25.4)
Episodes with associated 14-day mortality^*f*^	45 (36.9)

a Episodes, including 25 multiple episodes from 20 patients, were identified using standard-of-care (SoC) testing methods (see text for details).

b Includes 3 polymicrobial infections caused by both on-panel and off-panel organisms, such as Staphylococcus aureus and Hafnia alvei (1 episode), Klebsiella pneumoniae and Morganella morganii (1 episode), or K. pneumoniae, Pseudomonas aeruginosa, and Stenotrophomonas maltophilia (1 episode).

c Includes 2 infections caused by S. maltophilia and Citrobacter koseri (1 episode) or by S. maltophilia (1 episode).

d Antimicrobial resistance (AMR) of causative organisms was assessed by phenotypic methods and/or PCR-sequencing analysis. With respect to AMR-associated genes, 53 *bla*_oxa-23_ genes (all detected in Acinetobacter baumannii organisms) were off-panel targets, whereas two *mecA*/-*C* (and MREJ) genes (all detected in Staphylococcus aureus organisms) were on-panel targets but the relevant organisms did not grow in culture (see Table 1 for details).

e Bacteremia occurred within ±2 days of bacterial pneumonia diagnosis.

f Death was attributed to bacterial pneumonia in only 30 (66.7%) of 45 episodes. In the remaining episodes (15/45, 33.3%), death could be attributed to causes other than bacterial pneumonia (e.g., cerebral hemorrhage, heart failure, nonconcurrent bacteremia, etc.).

As shown by the results in Table 2, 77 (63.1%) and 45 (36.9%) of 122 episodes (78 of which were caused by antimicrobial-resistant organisms) were from patients who survived or did not survive, respectively, within 14 days of receiving FA-PP panel-targeted antimicrobial therapy. Twenty-eight (35.9%) of 78 antimicrobial-resistant episodes were from patients who did not survive bacterial pneumonia (22/28) or had another cause of death (6/28). Among 22 deadly episodes, colistin-resistant A. baumannii caused 9 episodes, MRSA caused 5 episodes, CTX-M-positive E. coli caused 3 episodes, and KPC-positive, CTX-M-positive K. pneumoniae caused 2 episodes. In these episodes, patients were treated with antibiotics deemed to cover all but A. baumannii antimicrobial-resistant organisms. Nonetheless, based on their recent clinical efficacy (15), we appropriately adopted fosfomycin-containing (triple-combination) regimens to treat colistin-resistant A. baumannii pneumonia episodes in our study (Fig. 2).

### Impact of FA-PP results on bacterial pneumonia episode outcomes.

We additionally studied a cohort of patients (*n = *29) with bacterial pneumonia who had been hospitalized in our ICU before the FA-PP panel was implemented. For these patients, a documented bacterial pneumonia etiology was exclusively possible by means of SoC methods. Comparing their clinical characteristics with those of patients from the current study cohort (*n = *97) showed no statistically significant differences for most of them, including for 14-day mortality (Table 3). Interestingly, in 6 (19.3%) of 31 episodes, patients were receiving effective antibiotic treatment within 6 h from LRT sampling (which corresponded to the averaged time an FA-PP panel result could be obtained), compared to 105 (86.1%) of 122 episodes for which an FA-PP panel result was actually obtained (*P* < 0.001).

**TABLE 3:** Demographics and clinical characteristics of COVID-19 patients diagnosed with bacterial pneumonia during current or previous ICU stay periods^*a*^

Characteristic	Value for patients with pneumonia episodes from the:		*P* value
	Current period (*n *= 97 patients; *n *= 122 episodes)	Previous period (*n *= 29 patients; *n *= 31 episodes)	
Age (yrs) [median (IQR)]	67 (60–72)	65 (59–73)	0.96
Male [no. (%)]	76 (78.4)	27 (93.1)	0.09
Severity of illness at time of diagnosis [median (IQR)]			
SOFA score	5 (4–6)	4 (3–6)	0.09
SAPS II	38 (29–56)	38 (33–47)	0.84
Length of stay in ICU (days)	9 (5–17)	18 (5–34)	<0.001
Duration of mechanical ventilation (days)	7 (1–13)	10 (4–19)	0.07
Laboratory findings at time of diagnosis [median (IQR)]^*b*^			
Procalcitonin level (ng/ml)	0.38 (0.14–0.91)	0.45 (0.11–2.2)	0.77
C-reactive protein level (mg/dl)	150.5 (88.3–206.3)	127.0 (47.8–188.2)	0.10
Serum ferritin level (ng/ml)	986 (619–1641)	1125 (501–2687)	0.35
WBC count (×10^9^/liter)	14.2 (9.8–19.1)	13.2 (6.1–18.8)	0.29
Neutrophil count (×10^9^/liter)	12.6 (7.8–16.4)	10.5 (5.2–17.3)	0.41
Clinical outcomes [no. with outcome/total no. (%)]			
14-day mortality	45/122 (36.9)	10/31 (32.3)	0.41
28-day mortality	73/122 (59.8)	12/31 (38.7)	0.05
Effective antibiotic receipt within 6 h from respiratory tract sampling	105/122 (86.1)	6/31 (19.3)	<0.001

a For comparison purposes, we included patients who had been hospitalized in the ICU during the first wave of COVID-19. We used the Mann-Whitney *U* test or Fisher’s exact test to analyze continuous (expressed as median with IQR) or categorical (expressed as number with percentage) variables between the two patient groups. These periods represent the periods with FA-PP panels implemented (current period, 24 September 2020 to 8 March 2021) or not implemented (previous period, 23 March 2020 to 30 June 2020) in routine clinical use. Exclusively using standard-of-care culture methods, Staphylococcus aureus, Pseudomonas aeruginosa, and Klebsiella pneumoniae were the most common etiological agents of bacterial pneumonia identified in the previous period studied. COVID-19, coronavirus disease 2019; ICU, intensive care unit; IQR, interquartile range; SOFA, sepsis-related organ failure assessment; SAPS II, simplified acute physiology score II; WBC, white blood cells.

b For each biological parameter listed, level/count abnormality was assessed based on the reference value/range. The ranges were set as follows: procalcitonin, ≤0.1 ng/ml; C-reactive protein, ≤6 mg/dl; serum ferritin, 10 to 291 ng/ml; WBC, 4 × 10^9^ to 11 × 10^9^ cells/liter; and neutrophils, 2.0 × 10^9^ to 7.5 × 10^9^ cells/liter.

The costs for testing LRT samples with the FA-PP panel in the current patient cohort amounted to ∼56,604 euros (i.e., 267 euros [the cost of a single test] × 212 samples), whereas costs in the previous patient cohort would amount to 36,846 euros (i.e., 267 euros × 138 samples [including 107 samples with SoC negative results]). In the current patient cohort, costs were counterbalanced by the timely benefits of either preventing unnecessary antibiotic treatment in 80 (88.9%) of 90 cases with SoC-negative LRT samples or appropriately treating 115 (94.3%) of 122 cases with SoC-positive LRT samples (Fig. 2).

## DISCUSSION

We showed the practicability of FA-PP panel results for accurate diagnosis, with 100% PPA and 96.4% to 100% NPA with SoC culture results, for 202 bacterial species detected in total and prompt management of antimicrobial therapy in 120 (98.4%) of 122 bacterial pneumonia episodes that were positive for one or more on-panel organisms. Two polymicrobial episodes were positive for off-panel organisms (S. maltophilia or Hafnia alvei) that were not covered by the (empirically administered) antimicrobial therapy at the time of FA-PP panel testing. Excluding these episodes, FA-PP panel results allowed initiating or changing organism-targeted antibiotics in 118 (98.3%) of 120 episodes. Overall, antimicrobial therapy interventions occurred within times (∼6 h, on average) from LRT sampling that were considerably shorter than times (∼72 h, on average) when culture results would have been actionable for intervention.

### Antimicrobial stewardship according to FA-PP results.

In addition to confirming its diagnostic accuracy (12–14), we showed that the FA-PP panel contributed concretely to antimicrobial stewardship in COVID-19 patients with bacterial pneumonia. Implementing the FA-PP panel at our institution resulted in 101 (84.2%) of 120 episodes for which patients received appropriate (AST-confirmed) antibiotic therapy, as assessed for one or more FA-PP-detected organisms. In patients who were empirically treated when their LRT samples were obtained, FA-PP results enabled ICU physicians to tailor (i.e., escalate or deescalate) or stop (i.e., discontinue) antibiotics as soon as possible. This timing was coincident with the time an FA-PP result was available, which was ∼66 h earlier than the SoC result time for patients whose samples were concordantly FA-PP/culture positive.

Overall, antibiotics were discontinued in 33.3% of patients whose samples yielded a negative FA-PP result, whereas antibiotic therapy was modified or left unmodified in 100% of patients whose samples yielded a positive FA-PP result. In particular, therapeutic interventions involved 19 patients whose samples yielded additional FA-PP-detected organisms, for which antibiotic coverage was necessarily broadened. Consistently, in four cases, patients were treated with antistaphylococcal or anti-P. aeruginosa antimicrobial agents, but inappropriately if we consider the culture result as the “ground truth.” Conversely, patients whose samples yielded organisms only detected by culture (two of which were S. maltophilia) received appropriate antibiotic treatments, which would have been initiated earlier if these organisms had been included among the FA-PP panel targets. Therefore, the risk of overusing antibiotics was counterbalanced by the risk of delaying the delivery of effective antibiotics to our ICU patients.

It is noteworthy that many patients with FA-PP-negative samples were still treated. Therefore, in some cases, pneumonia was still suspected clinically, suggesting that a negative LRT sample result does not necessarily rule out pneumonia (16). Unfortunately, decisions to continue to treat patients may potentially increase the emergence of multidrug-resistant pathogens, such as P. aeruginosa, Acinetobacter spp., and MRSA (17).

### Microbiological etiology according to FA-PP results.

It has been noted that most of the bacterial organisms detected in LRT secretions from COVID-19 patients within the first 10 days of mechanical ventilation are community pathogens with minimal AMR profiles (8; unpublished data). This finding would apparently contrast with the findings reported by others (3, 4, 7, 18) or us (19; this study), which document the predominance of nosocomial pathogens as causes of bacterial pneumonia in critically ill patients with COVID-19 (20). Overall, approximately 72% of our pneumonia episodes were caused by A. baumannii and/or S. aureus. In one study (18), approximately 50% of bacterial pneumonia in patients with severe COVID-19 requiring mechanical ventilation was due to S. aureus. In another study (1), S. aureus accounted for 69.2% of early bacterial coinfections in ICU patients with COVID-19-related acute respiratory distress syndrome, and all organisms were methicillin susceptible. Here, among bacterial pneumonia-causing organisms, 49.0% of S. aureus organisms were MRSA, and 100% of A. baumannii organisms were carbapenem resistant. This finding is in line with ever-consolidating concepts that, while in critically ill patients with COVID-19, the prevalence of bacterial superinfections remains unclear (21), the risk of bacterial superinfections (particularly [multi]drug-resistant infections) is higher the longer the patient’s stay under mechanical ventilation or in the ICU (20). Thus, as justly noted by Bassetti et al. (21), it is not surprising that the major culprits were Gram-negative bacterial organisms in some studies and Gram-positive bacterial organisms in other studies. The rate of multidrug-resistant A. baumannii was quite high in our study, thereby justifying the routine use of the FA-PP panel for antibiotic escalation purposes. Unfortunately, the usefulness of the FA-PP panel may be more limited in hospital (ICU) settings with lower rates of A. baumannii infection or if considering non-COVID-19 patients. Only six A. baumannii VAPs from these patients were diagnosed in our study period (data not shown).

### Strengths and limitations.

Since the pre-COVID-19 era, bacterial pneumonia diagnosis has been in the spotlight, leading the MagicBullet clinical trial group to show the usefulness of the BioFire FilmArray blood culture panel. No BioFire FilmArray pneumonia panel was available at that time (22) to rule out VAP-causing organisms, especially multidrug-resistant Gram-negative organisms, in patients from European hospitals, including ours (23). To the best of our knowledge, the present study adds to a few published studies about FA-PP panel use in the ICU/COVID-19 setting (10, 11, 24). Similar to Maataoui et al.’s study (24) but unlike other authors’ studies (10, 11), we focused primarily on how FA-PP panel results could support antimicrobial therapeutic decisions for bacterial pneumonia in COVID-19 patients hospitalized in an ICU. Here, we did not conjecture about antimicrobial stewardship interventions in our ICU patient cohort, as previously done (23), but we performed such interventions to comply with well-established antibiotic stewardship program efforts in the ICU (25).

However, this study is not without limitations. We roughly explored the effect of FA-PP panel-driven therapeutic decisions on ICU mortality by calculating the proportions of patients who were deceased or still alive since the administration of 14 days of potentially effective antibiotics. Therefore, we did not discuss this issue further because our study design prevented us from drawing firm conclusions. However, an additional comparative analysis of bacterial pneumonia patients from two different COVID-19 epidemic waves to represent the periods with or without implementation of the FA-PP panel in routine clinical use allowed us to show that FA-PP panel use might actually result in timely administration of effective antibiotics. Second, our study was limited not only by its monocentric nature but also by the fact that the study period, contrary to other studies (10, 11, 24), was coincident with the second wave of the COVID-19 epidemic. This timing reduces both the generalization and comparability of the study findings. Third, VAP is much more than the outcome of a single pathogen entering the lung and then causing infection (26), and its complex pathophysiology may shadow the relevance of commonly detected bacterial pathogens. Fourth, some patients were already on antibiotics when LRT samples were obtained for microbiological testing. This situation implied that SoC culture-negative results might be false-negative results, thus biasing our assessment of FA-PP panel performance. Fifth, we did not include data about the weekly assessment of nasal or rectal colonization status for the patients studied. Consequently, we did not know whether this information might have influenced the appropriateness of empirically administered antibiotic treatments in our patient cohort.

### Conclusions.

Our experience with the FA-PP panel shows that using molecular-panel-based assays for the detection and quantification of bacterial etiologies (and their AMR profiles) in LRT samples may become indispensable for the clinical and therapeutic management of VAP or non-VAP episodes in ICU patients with COVID-19. Future prospective trials are needed to understand whether FA-PP panel use on a large scale may effectively contribute to improving the health of COVID-19 patients.

## MATERIALS AND METHODS

### Ethics.

This study was approved by the Ethics Committee of our institution (approval number 17057/20). The requirement for written informed consent was waived because testing of patients’ samples and ensuing therapeutic decisions were part of routine clinical care.

### Study design, samples, and data.

We studied LRT (BAL fluid or ETA) samples that were prospectively collected between 24 September 2020 and 8 March 2021 from patients with a COVID-19 diagnosis (i.e., a SARS-CoV-2 infection confirmed via nasopharyngeal swab reverse transcription [RT]-PCR assay) (27) who were admitted to the ICU of the Fondazione Policlinico Universitario A. Gemelli IRCCS tertiary-care university hospital in Rome, Italy (Fig. S1). We included samples that were obtained from all mechanically ventilated patients at the time of suspicion of developing pneumonia, which relied on the criteria of the current American Thoracic Society/Infectious Diseases Society of America (ATS/IDSA) (28) or European Centre for Disease Prevention and Control (ECDC) (29) HAP/VAP guidelines. As specified elsewhere (2, 4), the criteria included a combination of fever (body temperature of ≥38°C), leukocytosis (white blood cell count of ≥10 × 10^9^/liter), purulent tracheal secretions, new or progressive infiltrates on chest radiograph, and deterioration of blood gas exchange. In all cases, according to Papazian et al. (16), at least two of these criteria were satisfied, resulting in 11.3% (24/212), 38.2% (81/212), 34.4% (73/212), 69.8% (148/212), or 26.9% (57/212) of samplings in which the patients had fever, leukocytosis, purulent secretions, infiltrates, and/or gas exchange deterioration, respectively. There were only five cases with LRT samples whose semiquantitative cultures (see below) provided nonclinically relevant bacterial concentration values (e.g., <10^5^ CFU/ml for an ETA sample) and were excluded from the study. We classified pneumonia episodes as VAP or non-VAP if patients were receiving mechanical ventilation for >48 h or ≤48 h, respectively, at the time of diagnosis (9). In patients with multiple episodes, VAP was defined as a relapse if the initial causative bacterial pathogen(s) (i.e., same species and AMR profile) grew at a clinically relevant concentration (e.g., ≥10^5^ CFU/ml for an ETA sample) from the subsequently collected sample. Otherwise, VAP was considered a new episode.

Study data included demographics (e.g., age) and illness severity (e.g., simplified acute physiology score II), laboratory (e.g., procalcitonin), or outcome (e.g., mortality) data, which were collected by reviewing the medical charts of the bacterial pneumonia patients. These patients included not only ICU patients hospitalized in the period between 24 September 2020 and 8 March 2021 (when the FA-PP panel had been implemented) but also those hospitalized in the period from 23 March 2020 to 30 June 2020 (when the FA-PP panel had not been implemented). In the latter period, 138 LRT samples from the patients had been subjected to microbiology laboratory examination as described below. Substantially, we compared ICU patients from the second and third COVID-19 waves for characteristics like the receipt of effective antibiotics within 6 h from LRT sampling, which were likely affected by the timing at which an FA-PP panel LRT result was actionable for the patient’s antimicrobial treatment. We assessed mortality in terms of patients who died or lived at day 14 or 28 from LRT sampling.

### Microbiological testing.

In pneumonia episodes, microbiological evidence of bacterial infection was achieved by processing LRT samples both for SoC, including conventional Gram stain (performed on concentrated ETA or BAL fluid samples when necessary) and culture (30), matrix-assisted laser desorption ionization–time of flight (MALDI-TOF) mass spectrometry identification, and AST for aerobic bacteria, and for FA-PP panel testing. Gram staining results showed the presence of inflammatory cells in all samples, indicating that their collection had been performed appropriately (30). Semiquantitative culture results (expressed as CFU/ml) were obtained using calibrated loops to inoculate and streak samples on both selective/differential (blood, chocolate, MacConkey, and Columbia colistin-nalidixic acid [CNA]) and screening (chromID S. aureus elite [SAIDE; bioMérieux, Marcy l’Étoile, France] and chromID extended-spectrum β-lactamase [ESBL] [bioMérieux]) agar media. Screening agar medium was helpful to mitigate the confounding/masking effect of normal oropharyngeal microbiota on potential LRT pathogens. All plates were incubated in 5% CO_2_-enriched air and examined for growth at 24 to 48 h of incubation. Upon growth at or above 1 × 10^4^ CFU/ml for a BAL fluid sample or 1 × 10^5^ CFU/ml for an ETA sample, two commonly accepted thresholds of bacterial quantification that were assessed per morphotype, bacterial colonies were identified to the species level using MALDI-TOF mass spectrometry. The Vitek 2 system (bioMérieux) and/or the Micronaut broth microdilution panel (Merlin Diagnostika GmbH, Bornheim, Germany) were used to perform AST on the same colonies. In parallel, samples were tested using the FA-PP panel, which allowed automatic running (within approximately 75 min) of all steps to detect genomic sequences unique to each FA-PP panel target (12, 13). With this assay, semiquantitative results for 15 bacterial targets can be reported in log_10_ increments from 10^4^ to ≥10^7^ genome copies/ml, and results for seven AMR genes are reported as “detected” or “not detected” (12, 13). Cultures for fungi were also performed on LRT samples as part of SoC, but the results from these cultures were not included in this study. However, we noticed that two samples were positive for Aspergillus, which has emerged as a cause of superinfection in COVID-19 patients (31). Testing results were available from the LRT sample collection within an average time of 72 h for the SoC method or 6 h for the FA-PP panel method, comprising sample transport, processing, and testing times. The results were reported to ICU clinicians in real time according to the clinical microbiology laboratory’s standard operating procedure.

### Antimicrobial treatment and stewardship interventions.

Information about antimicrobial treatment (empirical or targeted), which relied on ICU regimens based on in-hospital culture patterns or antibiotic susceptibilities (25), was retrieved from patients’ medical charts. This review included the time(s) of antibiotic initiation or discontinuation, which was used to classify antibiotic therapy interventions (e.g., antibiotic escalation or deescalation) or no interventions (i.e., antibiotic therapy was left unmodified or not administered) according to FA-PP panel results, according to definitions reported elsewhere (13). The appropriateness of these interventions was definitively determined by comparing FA-PP panel results with the results of the SoC AST method. In particular, antibiotic escalations consisted of any broadening of antimicrobial coverage, which implied a change in agent (e.g., ceftriaxone to meropenem) or the addition of an agent(s) in a multidrug treatment. Antibiotic deescalations consisted of any narrowing of antimicrobial coverage, which implied a change in agent (e.g., meropenem to ceftriaxone) or the discontinuation of an agent(s) in a multidrug treatment. Consistently, antibiotic initiation or discontinuation was defined as the administration or the interruption of an agent(s), respectively, in patients who were not receiving or had previously received empirical therapy for suspected bacterial pneumonia. According to the number/type of organisms identified and/or of antimicrobials empirically administered, we classified some cases for more than one antibiotic therapy intervention. The difference between the time of antibiotic therapy intervention based on the FA-PP panel result and the time of antibiotic therapy intervention based on the SoC culture result was used to calculate the number of hours sooner that antibiotic(s) had been initiated or escalated.

### Data analysis.

Stata software version 11.1 (StataCorp, College Station, TX, USA) and GraphPad Prism version 7.0 (GraphPad Software, San Diego, CA, USA) were used to perform all statistical analyses. Using SoC testing as the reference method, we classified FA-PP panel (microbial species or AMR gene) detection results as true positive (TP), false negative (FN), false positive (FP), or true negative (TN). Then, we calculated positive percent agreement [PPA; TP/(TP + FN)] and negative percent agreement [NPA; TN/(TN + FP)] with their two-sided 95% confidence intervals (CI), determined according to the Clopper-Pearson method. With respect to the comparison of clinical characteristics in the two patient groups, as shown in Table 3, continuous (expressed as median values with interquartile ranges [IQR]) or categorical (expressed as numbers with percentages) variables were analyzed using the Mann-Whitney *U* test or Fisher’s exact test, respectively. Statistical significance was set at a *P* value of < 0.05.
