# Supplementary material for: Diagnosis and Treatment of Bacterial Pneumonia in Critically Ill Patients with COVID-19 Using a Multiplex PCR Assay: A Large Italian Hospital’s Five-Month Experience
Source: Microbiol Spectr. 2021 Nov 10;9(3):e00695-21. doi: 10.1128/Spectrum.00695-21 (PMC8579927; doi:10.1128/Spectrum.00695-21)

**FIG S1** Flowchart of patients and their lower respiratory tract samples included in the study. Standard-of-care testing (SoC) methods consisted of both culture-based identification and antimicrobial susceptibility testing, which were used as the reference method for comparison purposes.

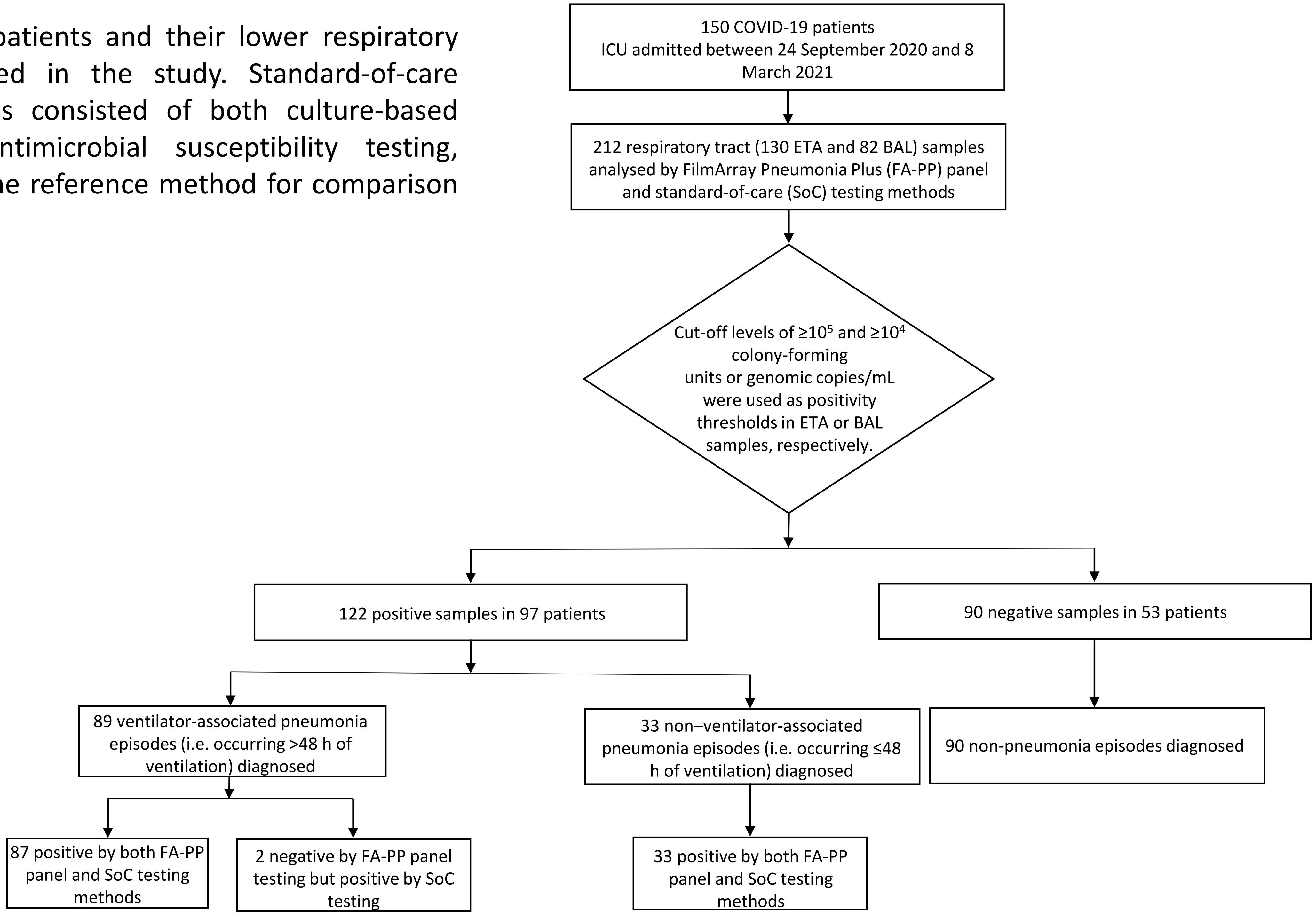

Supplement: SUPPLEMENTAL FILE 1 — Supplemental material. Download SPECTRUM00695-21_Supp_1_seq8.pdf, PDF file, 0.4 MB [file spectrum00695-21_supp_1_seq8.pdf]
